# Effect of the Post-Spinning Solvent Exchange on the Performance of Asymmetric, Polyimide Hollow Fibers Prepared by Using a Triple-Orifice Spinneret

**DOI:** 10.3390/ma12213632

**Published:** 2019-11-05

**Authors:** Paola Bernardo, Franco Tasselli, Giovanni Chiappetta, Gabriele Clarizia

**Affiliations:** Institute on Membrane Technology, ITM-CNR, c/o University of Calabria, Via P. Bucci 17/C, 87036 Rende (CS), Italy; p.bernardo@itm.cnr.it (P.B.); f.tasselli@itm.cnr.it (F.T.); g.chiappetta@itm.cnr.it (G.C.)

**Keywords:** hollow fiber membranes, triple spinneret, solvent–exchange, gas separation, polyimide

## Abstract

Hollow fibers (HFs) are widely applied in different membrane operations, particularly in gas separation. The present work investigates the effect of post-spinning treatment on the gas transport properties of polyimide-based HFs. The membranes were spun by using both a conventional spinneret and a triple-orifice spinneret. A systematic analysis was carried out by considering different alcohols as the first fluid for the solvent exchange, with or without *n*–hexane as a second fluid. The HFs were characterized by exploring the change of the morphology and the permselective properties as a consequence of the operation conditions for spinning and post-treatments. According to the morphology, for a specific hollow fiber type, an optimal post–treatment was identified. The HFs prepared with the triple-orifice spinneret, using a solvent–rich shell fluid, can take advantage of the post-treatment using larger alcohols, while smaller alcohols should be preferred for the conventional spun HFs that present inside–outside double skin layers.

## 1. Introduction

Polymeric materials, such as polyimides [1], play an important role in membrane gas separation. These materials can be shaped as hollow fibers (HFs), which is the most used configuration in this field owing to its superior membrane packing density, leading to compact devices [2]. The small physical footprint of membrane modules is typically associated with low energy requirements [3]. Therefore, membrane systems are increasingly considered as appealing alternatives to conventional gas separation methods, such as cryogenic distillation or absorption [3,4]. An increasing number of plants are in operation for air separation, hydrogen recovery in refineries, natural gas sweetening, and biogas upgrading.

The spinning process to produce polymeric HFs provides different parameters (composition of dope and bore fluid, flow rates, air gap, temperature of dope and coagulation bath, take-up velocity) that can be varied in order to modify the membrane structure, and thus, its gas transport properties [5]. The requirement of higher permeation fluxes to enhance productivity lead towards asymmetric structures. These present a thin skin layer, responsible for the permselective properties, onto a macroporous support that guarantees the necessary mechanical resistance to operate up to a few dozen bars. The phase inversion technique is ideally suited to produce such asymmetric structures. However, in the case of these structures, prepared by the dry–wet phase inversion method, the non-solvent (generally water) evaporation during the drying step results in capillary forces that generate a thickening of the skin layer and the collapse of the transition layer [6]. Accordingly, the HFs become denser and less permeable. Therefore, the solvent exchange process is critical to prevent the pore structure from collapsing during drying, and is typically adopted for porous membranes. Some modification methods were specifically developed for the HF post-spinning treatment, in order to preserve the original microstructure and prolong its lifetime. A similar approach was applied to convert reverse osmosis membranes to gas separations membranes [7]. The solvent exchange protocol involves a polar fluid, such as an alcohol, to first wash out and replace the original non-solvent (water); this is followed by a low-surface-tension fluid, such as a hydrocarbon, to wash out the polar fluid. In principle, the evaporation of the second solvent, having a low surface tension, keeps the pore structure.

Clausi and Koros applied a solvent exchange procedure for gas separation, Matrimid-based HFs, rinsing the as-spun HFs in water, then in methanol, and finally in *n*-hexane [8]. In their study, the solvent exchange led to extremely permeable HFs, even if the selectivity for small/large molecules (e.g., He/N_2_) was somewhat depressed. This procedure is reported for asymmetric polyimide membranes to be applied in gas separation [9,10] or pervaporation [11]. The main limit of this protocol is its apparent generality, irrespective of the actual membrane structure and its final use.

Our previous work [12] evidenced the advantageous use of a triple spinneret for tailoring the structure of the HFs for gas separation. The feeding of an external fluid to the triple-orifice spinneret is a proper approach to modify the gas transport properties, eliminating the transport resistance located on the outer layer, thus leading to improved permeance and selectivity.

In the present paper, very permeable, asymmetric polyimide HFs were prepared by dry-jet/wet-quench spinning, using both a double-orifice and triple-orifice spinneret, in order to study structures with different morphologies. HFs prepared with a conventional spinneret have a double skin layer structure, while single skin layer structures are achievable by a triple-orifice spinneret. The HFs were post-treated to enhance their gas permeation properties, systematically investigating the effects of the procedure on the HF performance and how these effects differ when changing the HF morphology. Different solvent exchange fluids were adopted in order to take advantage of a “template effect” on the final membrane microstructure. They include methanol, ethanol, *i*-propanol 1-butanol, or *tert*–butyl alcohol as the first fluid and *n*-hexane as the second fluid.

## 2. Materials and Methods

**Figure 1:**
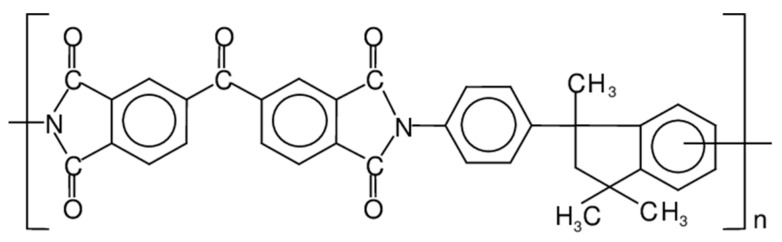
Repeat unit of the Matrimid polyimide.

The polyimide 3,3′,4,4′-benzophenonetetracarboxylic dianhydride and diaminophenylindane (Matrimid 5218, Figure 1) was supplied by Huntsman Advanced Materials (Belgium). N-methyl-2-pyrrolidone (NMP; purity of 99%) was purchased from VWR International (Italy). Methanol, ethanol, *i*-propanol, 1-butanol, *tert*-butanol and *n*-hexane (VWR International, Italy) were used for the solvent exchange procedure. The chemicals were used as received. Single gas permeation rate tests were carried out with CO_2_, He, H_2_, N_2_, O_2_, and CH_4_, (purity of 99.99%; SAPIO, Italy). A bi-component epoxy resin (Elan Tech, supplied by ELANTAS Italia S.r.l., Italy) was used for potting the HFs in the modules used for testing.

### 2.1. Membrane Preparation

The hollow fiber membranes were prepared according to a dry-jet/wet-quench spinning process in a spinning pilot plant described elsewhere [13]. The triple spinneret has nominal dimensions of 1.3/0.8/0.4 mm for outer, inner, and needle diameters, respectively [12]. Dope solutions were prepared by dissolving the polyimide in NMP at a concentration of 24 wt %. The polymer powder was slowly added to the solvent into a glass flask under mechanical stirring. The solution was left at 50 °C overnight under mechanical stirring (ca. 300 rpm). The spinning process was carried out at room temperature, with the dope kept at 50 °C. Deionized water was used as coagulation medium; tap water was used for rinsing the HFs during the collection on a reel.

Three HF batches were prepared. The operating conditions for spinning are listed in Table 1. The reference HFs (M1) were prepared without a shell fluid, thus using the triple orifice spinneret as a conventional double orifice spinneret. The M2 and M3 samples were spun using a solvent-rich shell fluid sent through the spinneret on the outer side of the fiber; this was done between the spinneret exit and the water bath in the dry-jet step. Deionized water was used as bore fluid, in order to guarantee a strong coagulation on the inner side. In particular, an appropriate air gap was selected to take advantage of the triple-orifice spinneret, with an adequate contact time for the external fluid and the nascent HF. Typically, a large air gap induces greater elongational stresses on polymer chains, thus resulting in denser skin layers [14].

### 2.2. Membrane Solvent Exchange

The as-spun hollow fibers were left in deionized water for two days at room temperature, in order to ensure the solvent–nonsolvent exchange, with a thorough rinsing using fresh deionized water daily.

Table 2 summarizes the physical properties of the used solvent exchange fluids. In addition, the Hansen solubility parameters [15] for the polymer and the fluids are provided. The reported empirical parameter *E*_T_(30) describes the polarity of the solvents.

The protocols adopted for the post-spinning treatments are defined in Table 3. Some HFs were dried from the water–wet state at room conditions (Protocol #1). The combined alcohol/*n*-hexane treatments are identified as Protocols #2–4. Other HFs were treated only with alcohols (i.e., methanol, ethanol, *i*-propanol, *tert*-butanol, or 1-butanol) before drying (Protocols #5–9). For each fluid, the procedure involved repeated baths with fresh fluids (three baths of 20 min each).

### 2.3. Membrane Characterization

The morphology of the prepared HFs was observed by means of scanning electron microscopy (SEM) analysis. The fibres were crio-fractured and sputter-coated with a thin film of gold. Sample images were acquired on an EVO|MA 10 (Zeiss, Milan, Italy) microscope, working in high-vacuum mode operated at 20 kV using secondary electrons.

### 2.4. Gas Permeation Tests

The permeation properties on the prepared HFs were evaluated in a fixed volume/pressure increase instrument (Elektro & Elektronik Service Reuter, Geesthacht, Germany) using single gases [18]. The system is comprised of a turbo molecular pump, with a membrane backing pump for the evacuation of the samples. When the membrane is exposed to the feed gas, a pressure transducer records the pressure increase in the permeate volume. The gas permeance, ∏, can be obtained at steady state conditions from the slope of the equation that describes the permeate pressure as function of time. The ideal selectivity was determined as the ratio of pure gas permeance coefficients.

HF samples were potted in small lab scale modules, and were closed at the other end with a drop of epoxy resin. The module was mounted inside a stainless-steel pressure cell for the gas permeation rate measurements, feeding the gas on the lumen side. The active HF length was approximately 10 cm. The tests were carried out at 25 °C and at a feed pressure of 1 bar, with the gases being tested according to the following order: H_2_, He, O_2_, N_2_, CH_4_, and CO_2_.

## 3. Results

The protocols adopted for the post-spinning treatments are described in Table 3.

**Figure 2:**
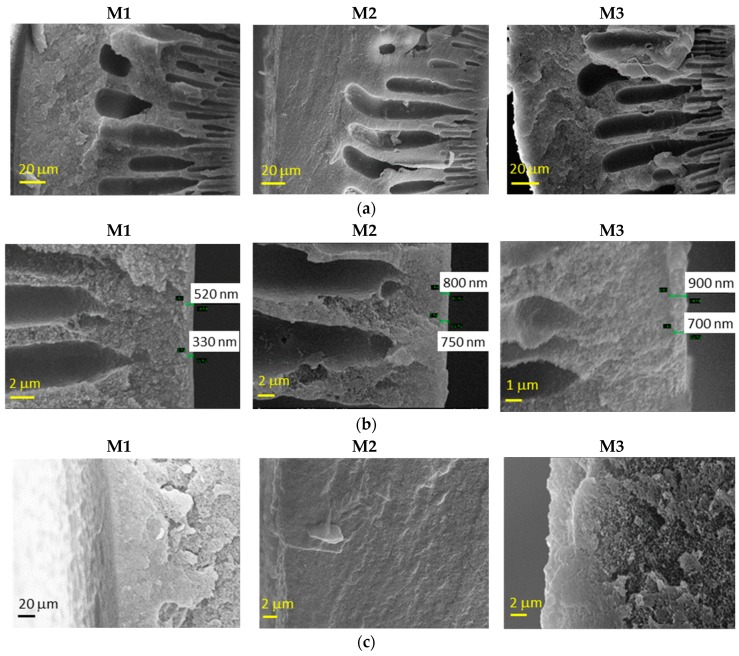
Representative scanning electron microscopy (SEM) images of Matrimid-based HF membranes dried with Protocol #1: (**a**) whole cross-section, (**b**) inner edge of the cross-section and (**c**) outer edge of the cross–section.

The morphology of the HFs dried according to Protocol #1 is shown in Figure 2. The fibers presented a typical asymmetric structure, with a nearly dense inner skin layer and a porous substructure. The HFs prepared without the external fluid in the dry-jet stage (M1) also presented a dense layer on the external side, due to the use of water as an external coagulant. Instead, when a solvent-rich external fluid was used in the air gap, the external spongy layer was less compact (M2 and M3), without the formation of a dense skin. Indeed, a delayed de-mixing occurs, resulting in a porous external surface [12]. In general, SEM images demonstrated that slight differences are present in the M2 and M3 HF batches. The greater the spinning velocity (M2), the thinner the skin thickness is.

**Figure 3:**
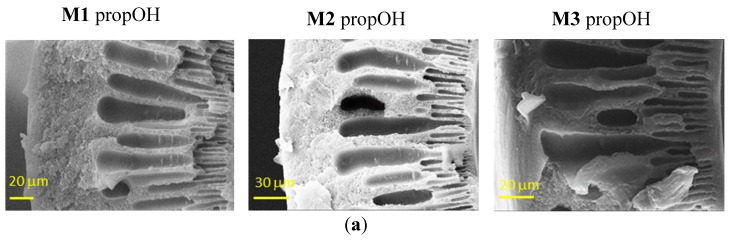

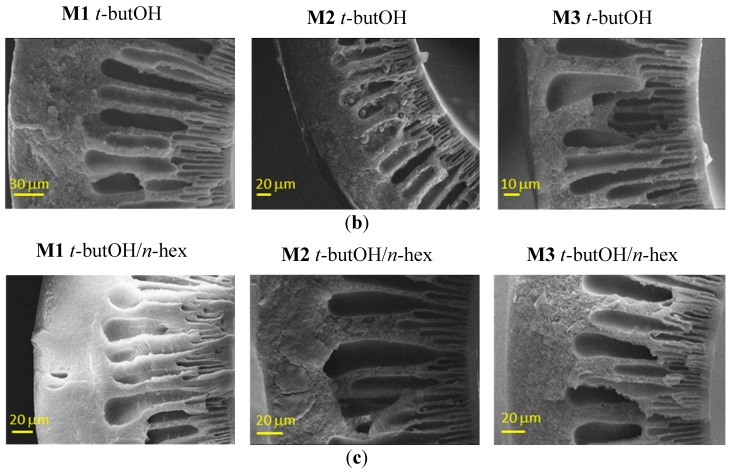
Representative SEM images of the cross-sections of Matrimid-based HF membranes dried with Protocol #7 (**a**), Protocol #8 (**b**) and Protocol #4 (**c**).

Figure 3 shows the effect of the post-treatments on the HFs. In general, the morphology seen in Figure 2 is preserved, but the contact with the fluid during the solvent exchange induced a partial damage of the skin layer that is more important in the case of denser structures.

**Figure 4:**
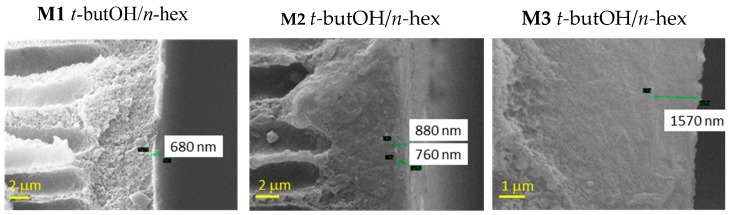
Representative SEM images of Matrimid-based HF membranes dried with Protocol #4. Inner edge of the cross–section.

A swelling of the inner side can be appreciated from Figure 4, in the case of the combined *t*-butOH/*n*-hexane treatment, particularly on the M3 samples.

The single gas permeation experiments, owing to the small molecular size of the tested penetrants, proved the effect of the operating conditions on the membrane microstructure and performance. Table 4 reports the permeation parameters of the asymmetric HF samples, treated according to Protocol #1 and dried in air. These data represent a reference for the changes produced by the different protocols adopted in this work. As usual in polyimides, hydrogen was the most permeable species. The selectivity was adequate, indicating nearly defect-free dense skin layers, with no need for post-treatment defect repairs (e.g., silicone coating). In general, the apparent transport parameters of the asymmetric membranes depend on the skin layer, but are also affected by the porous substructure [8]. The M1 HFs, spun without a shell fluid, evidenced a permselectivity lower than the intrinsic values quoted on Matrimid-dense sheets [12], but larger than values measured on other HFs based on the same material [19]. Instead, the M2 and M3 HFs, prepared with a solvent-rich solution (NMP/water 95/5) as shell fluid, were more permeable than the M1 HFs spun in a traditional double-orifice spinneret and highly selective membranes when tested after drying from the wet state. These data confirm the beneficial effect of a solvent-rich solution as the external fluid, in order to suppress the transport resistance located on the HF outer side [12]. In these samples, the inner skin layer governs the gas transport: its integrity affects the gas selectivity, while its thickness determines the gas permeance. The M2 HFs present gas transport properties close to the intrinsic values reported for Matrimid-dense films [12], while the M3 HFs have intermediate permeance and selectivity values relative the M1 and M2. In fact, a slightly thick skin layer was obtained for the M3 samples, as observed from SEM analysis. The comparison of M2 and M3 shows a larger dope extrusion rate (M2), leading to a better permeance and selectivity by enhancing polymer chain alignment and packing, resulting in the shift and sharpening of the free volume distribution [20].

The post-treatments (Protocols #2 and #5) on HFs that were dried in air (Protocol #1) did not increase the gas permeance. Therefore, after drying in air from the wet state, the HF asymmetric structure is well consolidated. On the contrary, the solvent exchange carried out on the wet HFs was able to modify their structure, and thus, the gas permeation parameters.

**Figure 5:**
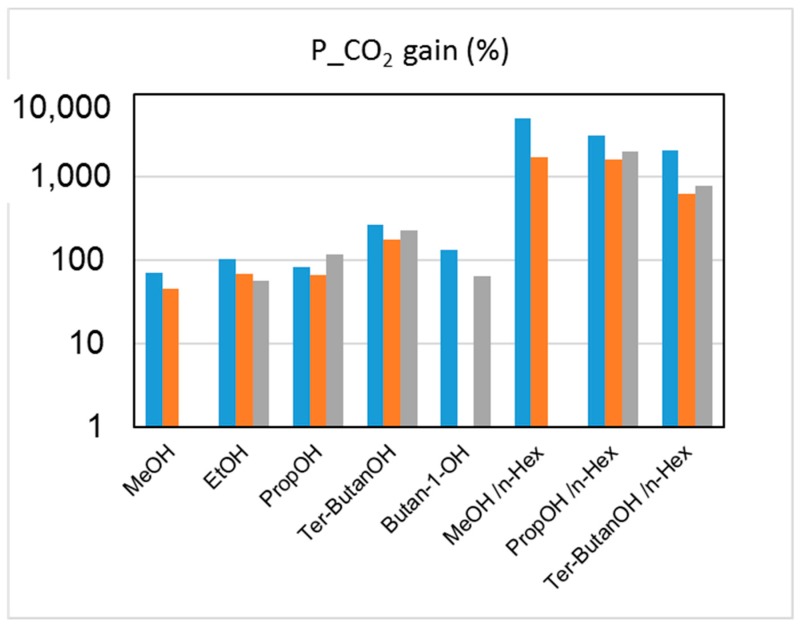
Permeance increase (%) for CO_2_ upon post-treatment (Protocols #2–9), relative to the data obtained after Protocol #1 for the three HF batches. Blue: M1; orange: M2; grey: M3.

The effect on the HFs’ performance of the solvent exchange protocol after the spinning can be examined by evaluating the changes induced on both gas permeance and selectivity. The capillary forces connected to the drying of HFs soaked in water (Protocol #1) induced a densification of the skin layer and a reduction of the pore sizes on the HF top layer, leading to a decreased gas permeance when compared to the other protocols. The permeance gain achieved via Protocols #2–9, relative to the data obtained after Protocol #1, is shown in Figure 5.

**Figure 6:**
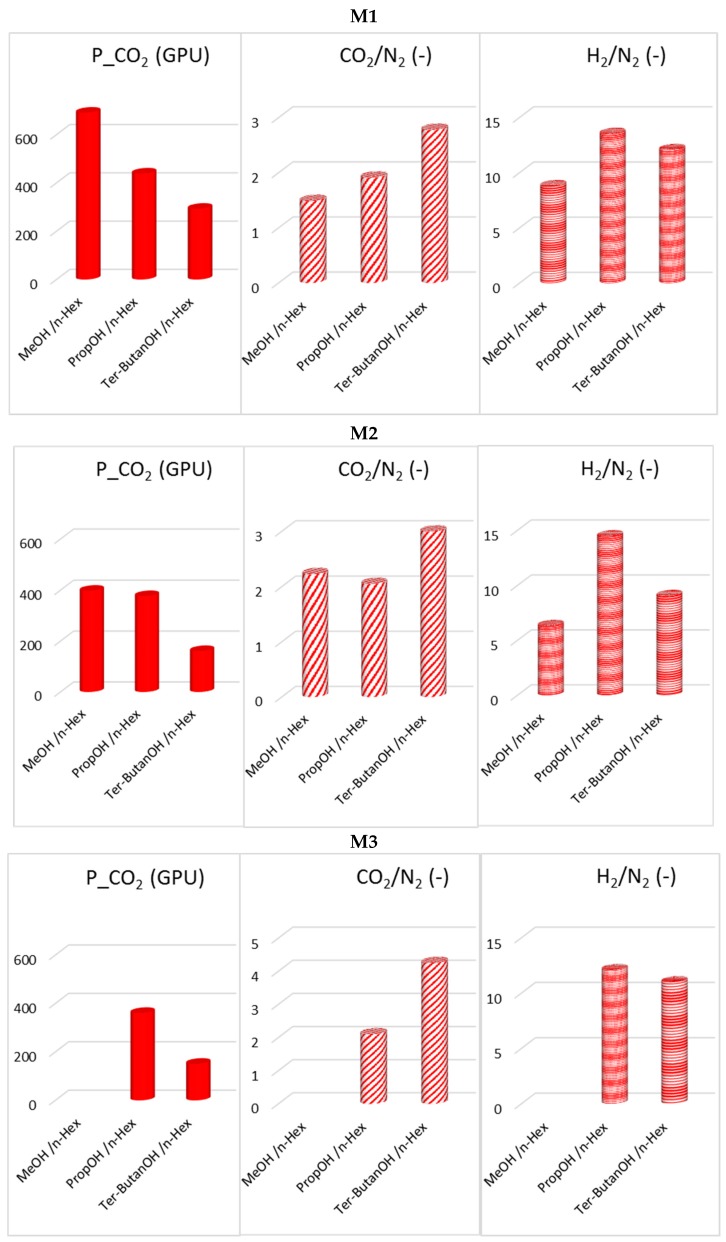
Post-treatment effect on CO_2_ permeance, as well as CO_2_/N_2_ and H_2_/N_2_ selectivity for M1, M2 and M3 HFs (Protocols #2–4).

**Figure 7:**
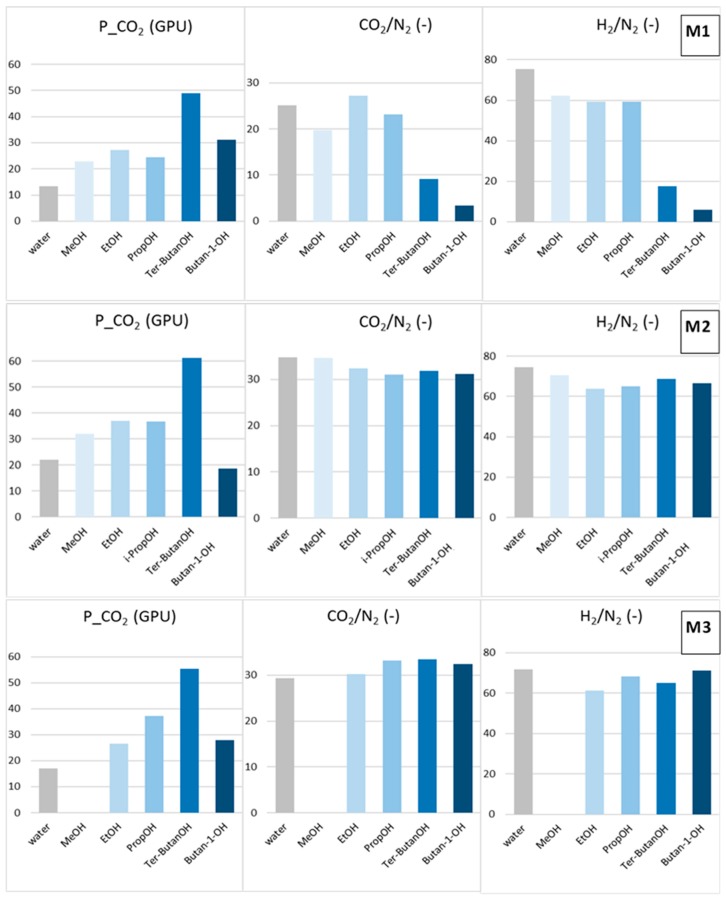
Post-treatment effect on the CO_2_ permeance, as well as CO_2_/N_2_ and H_2_/N_2_ selectivity for M1, M2 and M3 HFs (Protocols #5–9).

The combined alcohol/*n*-hexane treatments (Protocols #2–4) resulted in a permeance increase of more than one order of magnitude with respect to the data obtained after Protocol #1 (Figure 5). This is in agreement with what has been reported in the open literature [8]. However, the increase of permeance was obtained at the expense of permselectivity, which represents the other factor for evaluating the HF separating performance. Figure 6 and Figure 7 report CO_2_ permeance, as well as the CO_2_/N_2_ and H_2_/N_2_ selectivity as performance indicators of the membrane separating layer.

## 4. Discussion

The MeOH/*n*-hexane combination (Protocol #2) resulted in the largest permeability increase (M1 and M2). The gas permeation tests, evidencing a selectivity loss (trade-off between permeance and selectivity), revealed that this procedure compromises the integrity of the selective skin layer in the prepared HFs. Therefore, for the HF structures developed in this work, this procedure is not adequate when the ultimate application is in gas separation, while it was effective in the work of Clausi and Koros [8]. Indeed, the HFs prepared in this work, according to the spinning conditions described in Table 3, were already quite permeable when dried from a wet state (Protocol #1), if compared to the polyimide HFs described by Clausi and Koros [8]. A comparison of the HF permeance, with the permeability values typically quoted for Matrimid-dense films, indicates an apparent thickness of about 0.5 micron for the selective layer.

In general, in the combined treatments (Protocols #2–4), the permeance gain is reduced when using a larger alcohol, with an opposite effect on the CO_2_/N_2_ selectivity.

Looking at the solubility parameters gathered in Table 2, passing from water to an alcohol, the hydrogen bond term of the solubility parameter (*δ*_H_) and the polarity parameter decrease. Moving from methanol to *tert*-butanol, the alcohol polarity decreases, and there is better compatibility with *n*-hexane. The methanol/*n*-hexane combination (Protocol #2) presents a large difference for the *δ*_H_ term, while in the case of the *tert*–butanol it is reduced. Additionally, methanol and *n*-hexane have the largest difference in their molecular dimensions, while the lowest is found in the case of *tert*-butanol/*n*-hexane. The H_2_/N_2_ selectivity has a different trend than CO_2_/N_2_ selectivity (Figure 6), indicating a different HF microstructure with free volume elements of different sizes.

The solvent exchange involving only an alcohol (Protocols #5–9) avoided the selectivity loss evidenced in all the samples treated with the alcohol/*n*-hexane combination (Figure 7). These data indicate that the baths in *n*-hexane represent a critical phase in the two-step process. This treatment, owing to the swelling exerted by *n*-hexane, combined with rapid removal by evaporation leads to partial damage of the HF structure.

MeOH (Protocol #5) moderately increased both permeance and selectivity (samples M1 and M2) with respect to the Protocol #1. Bigger alcohols resulted in larger gas permeance, different from what observed in the combined treatments (Protocols #2–4). This trend could be related to a “template effect” exerted by the final solvent exchange fluid on the polymeric matrix. Indeed, a dependence of the transport properties on the molar volume of the solvent used for the membrane casting was reported for dense polysulfone membranes [21]. According to Kesting et al. [22], the solvent acts as a transient template (spacer) at the molecular level during the film formation process, reducing the packing density of the polymer chains. Internodular chain segment displacements are strongly affected by the size of the solvent molecules, which fold macromolecules in solution [21]. The alcohols selected for the present study are important protic solvents, displaying high polarity and high affinity with water. Accordingly, the imide groups present in the polyimide can strongly interact with them through polar–polar interaction and hydrogen bonding. These interactions are capable of altering the polymer chain packing density or chain mobility, as reported for polyimide membranes tested for sorption of alcohols [23].

Considering the gas permeance gain after Protocols #5–9, the best results were obtained with *tert*–butanol (Protocol #8) for all the HFs tested. The globular shape of *tert*-butanol is more suited for opening the polymer structure. In addition, *tert*–butanol has a low polarity and is more compatible with Matrimid, as can be seen from the close solubility parameters (Table 2). The 1-butanol (Protocol #9) represents an exception for all HFs. The longer 1-butanol molecule was not as efficient as *tert*-butanol in increasing the resulting permeation flux of the membrane, even if they are isomers. This behavior depends on the linear shape of the 1-butanol molecule, allowing its easy penetration inside the Matrimid polymer matrix, as evidenced in a pervaporation study carried out with different alcohols [10].

The analysis of the solvent effect on the different HF morphologies considered in this work showed that the post-treatment was more effective for the M1 HFs that presented inferior selectivity than for the intrinsic values of the polyimide when dried from the water–wet state (Table 4). In particular, ethanol was the best post-spinning treatment for the M1 HFs, also enhancing their selectivity, while *tert*–butanol and *i*–butanol improved the permeance but reduced the final selectivity. *Tert*-butanol and 1-butanol present solubility parameters more similar to those of the polymer; therefore, their use could damage the skins in M1 HFs. No particular effect on the selectivity of M2 HFs, which were already selective, was found when changing the alcohol type. In the case of the M3 HFs, the best combination of permeance and permselectivity was achieved using *tert*–butanol.

The observed differences depend on the structure of the samples: the prepared HFs present thin skin layers, but differ due to the presence of an outside dense skin (Figure 2). For HFs like M2 and M3, which have only an inner dense layer, the exchange procedure is facilitated and occurs through the porous external layer. Instead, for HFs like M1, the inside/outside double skin is swelled by the solvent exchange process, and is affected by the characteristics of the involved fluids.

In order to take advantage of the improved gas permeance obtained with the solvent treatment, even when the selectivity is depressed, it is always possible to combine it with a coating procedure (e.g., using a dilute silicone solution) that is typically and widely applied to seal small pinholes in membranes to be applied in gas separation.

## 5. Conclusions

Polyimide-based HFs, prepared by using a triple-orifice spinneret and a conventional spinneret, were treated post-spinning, using different fluids for the solvent exchange. The fibers have a typical asymmetric structure. Morphological analysis and gas transport measurements on the membranes dried directly from a wet state indicate the presence of defect-free skin layers.

The post-spinning treatment is appropriate to modify the HFs’ gas transport properties. The combination of an alcohol with *n*-hexane damages the selective skin layer, resulting in a permeance enhanced by more than one order of magnitude, with compromised selectivity. The permeation data indicate that the swelling caused by the *n*-hexane damages the structure of the prepared HFs. In this case, a healing protocol is necessary to use the membranes in gas separation.

For treatments involving only an alcohol, a larger molar volume of the alcohols increases the gas permeance of the HFs. The complex morphology of asymmetric hollow fibers results in different behaviors. In the case of HFs spun with a conventional spinneret, which have an inside/outside dense layer, lower alcohols are capable of increasing the permeance and the selectivity. The HFs obtained via a triple spinneret with a solvent-rich external fluid have a porous external surface. In this case, *tert*-butanol is the best choice for permeance gain and keeping the selectivity. This is due to the combination of a large molecular volume and a lower polarity of the alcohol, since the porous external layer facilitates the solvent exchange. Therefore, high-performance, asymmetric HFs with CO_2_/N_2_ selectivity higher than 30, as well as a CO_2_ permeance of ca. 60 GPU can be obtained, according to a solvent exchange protocol with *tert*-butanol.

## Figures and Tables

**Table 1:** Operating conditions for the preparation of the hollow fibers (HFs).

Conditions.	Membranes		
	M1	M2	M3
Dope flow rate (g min^−1^)	5	5	3.6
Bore Fluid (BF) flow rate (g min^−1^)	3	3	3
External Fluid (EF) flow rate (g min^−1^)	3	3	3
Air gap (cm)	60	60	60
Take-up velocity (m/min)	5.5	5.5	5.5
Temperature (*T*) dope (°C)	50	50	50
*T* coagulation bath (°C)	21	21	21
Bore fluid	water	water	water
Shell fluid	None	NMP/water (95/5)	NMP/water (95/5)

**Table 2:** Properties of the solvent exchange fluids used in this study.

Species	Molar Volume (mL mol^−1^)	Density (g mL^−1^)	Boiling Point (°C)	*δ*_D_ (MPa^1/2^)	*δ*_P_ (MPa^1/2^)	*δ*_H_ (MPa^1/2^)	*δ*_t_ (MPa^1/2^)	Polarity Parameter E_T_(30) ^c^ (kcal mol^−1^)
Water			100	15.5	16.0	42.3	47.8 ^a^	63.1
Methanol	40.5	0.791	64.7	15.1	12.3	22.3	29.6 ^a^	55.5
Ethanol	58.5	0.789	78.2	15.8	8.8	19.4	26.5 ^a^	51.9
*i*-propanol	76.8	0.803	82.3	15.8	6.1	16.4	23.6 ^a^	48.4
*tert*-butanol	92.1	0.805	83	15.2	5.1	14.7	21.8 ^a^	43.3
1-butanol	91.5	0.810	117.7	16.0	5.7	15.8	23.2 ^a^	50.2
*n*-hexane	131.4	0.659	69	14.9	0	0	14.9 ^a^	30.9
NMP	96.5	1.03	202	18	12.3	7.20	23.0	
Matrimid 5218		1.24	-	18.7	9.5	6.7	22.1 ^b^	

*δ*_D_, *δ*_P_, and *δ*_H_, are the dispersion, dipole, and hydrogen bonding terms of the solubility parameter *δ*_t_, respectively. ^a^ data from ref. [15]; ^b^ data from ref. [16]; ^c^ data from ref. [17].

**Table 3:** Protocols adopted for the solvent exchange procedure.

Protocol	Fluids for the Solvent–Exchange	HF Code		
		M1	M2	M3
Protocol #1	water	X	X	X
Protocol #2	water/methanol/*n*-hexane	X	X	-
Protocol #3	water/*i*-propanol/*n*-hexane	X	X	X
Protocol #4	water/*t*-butanol/*n*-hexane	X	X	X
Protocol #5	water/methanol	X	X	-
Protocol #6	water/ethanol	X	X	X
Protocol #7	water/*i*-propanol	X	X	X
Protocol #8	water/*t*-butanol	X	X	X
Protocol #9	water/*1*-butanol	X	X	X

**Table 4:** Permeation properties of the Matrimid HFs treated with Protocol #1. Temperature (*T*) = 25 °C.

Membrane	Permeance (GPU)						Selectivity	
	H_2_	He	CO_2_	O_2_	CH_4_	N_2_	CO_2_/N_2_	H_2_/N_2_
**M1**	40.0	33.5	13.3	3.35	0.44	0.53	25.1	75.5
**M2**	47.0	40.0	21.9	4.0	0.54	0.63	34.8	74.6
**M3**	41.6	37.0	17.0	3.6	0.52	0.58	29.3	71.7

GPU: Gas Permeance Unit. 1 GPU = 10^−6^ cm^3^·cm^−2^·s^−1^·cmHg^−1^

## References

[1] Ismail A.F., Jaafar J., Drioli E., Giorno L. (2016). Matrimid^®^ Membranes. Encyclopedia of Membranes.

[2] Mulder M. (2003). Basic Principles of Membrane Technology.

[3] Bernardo P., Drioli E., Golemme G. (2009). Membrane gas separation: A review/state of the art. Ind. Eng. Chem. Res..

[4] Bernardo P., Clarizia G. (2013). 30 years of membrane technology for gas separation. Chem. Eng. Trans..

[5] Alobaidy A.A., Sherhan B.Y., Barood A.D., Alsalhy Q.F. (2017). Effect of bore fluid flow rate on formation and properties of hollow fibers. Appl. Water Sci..

[6] Liu Y., Koops G., Strathmann H. (2003). Characterization of morphology controlled polyethersulfone hollow fiber membranes by the addition of polyethylene glycol to the dope and bore liquid solution. J. Membr. Sci..

[7] Minhas B.S., Matsuura T., Sourirajan S. (1987). Formation of asymmetric cellulose acetate membranes for the separation of carbon dioxide—methane gas mixtures. Ind. Eng. Chem. Res..

[8] Clausi D.T., Koros W.J. (2000). Formation of defect-free polyimide hollow fiber membranes for gas separations. J. Membr. Sci..

[9] Dong G., Li H., Chen V. (2010). Factors affect defect-free Matrimid^®^ hollow fiber gas separation performance in natural gas purification. J. Membr. Sci..

[10] Liu C., Bowen T.C., Harbert E.G., Minkov R., Faheem S.A., Osman Z. (2013). Polyimide Gas Separation Membranes. U.S. Patent.

[11] Jiang L.Y., Chung T.-S., Rajagopalan R. (2008). Dehydration of alcohols by pervaporation through polyimide Matrimid (R) asymmetric hollow fibers with various modifications. Chem. Eng. Sci..

[12] Bernardo P., Prete S., Clarizia G., Tasselli F. (2019). Effect of external fluid and inline crosslinking on the performance of polyimide hollow fibres prepared by using a triple–orifice spinneret. J. Membr. Sci..

[13] Tasselli F., Drioli E. (2007). Tuning of hollow fiber membrane properties using different bore fluids. J. Membr. Sci..

[14] Bernardo P., Tasselli F., Clarizia G. (2019). Gas separation Hollow Fiber Membranes: Processing conditions for manipulating morphology and performance. Chem. Eng. Trans..

[15] Hansen C.M. (2007). Hansen Solubility Parameters: A User’s Handbook.

[16] Loloei M., Moghadassi A., Omidkhah M., Amooghin A.E. (2015). Improved CO_2_ separation performance of Matrimid^®^ 5218 membrane by addition of low molecular weight polyethylene glycol. Greenhouse Gas Sci. Technol..

[17] Reichardt C. (1979). Empirical Parameters of Solvent Polarity as Linear Free-Energy Relationships. Angew. Chem. Int. Ed..

[18] Clarizia G., Bernardo P., Gorrasi G., Zampino D., Carroccio S.C. (2018). Influence of the Preparation Method and Photo-Oxidation Treatment on the Thermal and Gas Transport Properties of Dense Films Based on a Poly(ether-block-amide) Copolymer. Materials.

[19] David O.C., Gorri D., Nijmeijer K., Ortiz I., Urtiaga A. (2012). Hydrogen Separation from Multicomponent Gas Mixtures Containing CO, N2 and CO2 Using Matrimid Asymmetric Hollow Fiber Membranes. Procedia Eng..

[20] Ismail A.F., Ibrahim S.M., Nasri N.S.B. (2002). Effects of dope extrusion rate on the morphology and gas separation performance of asymmetric polysulfone hollow fiber membranes for O_2_/N_2_ separation. Songklanakarin J. Sci. Technol..

[21] Adewole J.K., Ahmad A.L., Ismail S., Leo C.P., Sultan A.S. (2015). Comparative studies on the effects of casting solvent on physico-chemical and gas transport properties of dense polysulfone membrane used for CO_2_/CH_4_ separation. J. Appl. Polym. Sci..

[22] Kesting R.E., Fritzsche A.K., Murphy M.K., Cruse C.A., Handermann A.C., Malon R.F., Moore M.D. (1990). The second-generation polysulfone gas-separation membrane. I. The use of lewis acid: Base complexes as transient templates to increase free volume. J. Appl. Polym. Sci..

[23] Qiao X., Chung T.-S. (2005). Fundamental Characteristics of Sorption, Swelling, and Permeation of P84 Co-polyimide Membranes for Pervaporation Dehydration of Alcohols. Ind. Eng. Chem. Res..

